# 基于全二维气相色谱-飞行时间质谱指纹图谱的原油溯源

**DOI:** 10.3724/SP.J.1123.2025.02005

**Published:** 2025-06-08

**Authors:** Weiya ZHANG, Pin CHEN, Weixin XIE, Xuanbo GAO, Wanfeng ZHANG, Wei DAI, Siyuan LIN, Shukui ZHU

**Affiliations:** 1.深圳海关工业品检测技术中心，广东 深圳 518067; 1. Testing and Technology Center for Industrial Products of Shenzhen Customs，Shenzhen 518067，China; 2.中国地质大学（武汉），地质微生物与环境全国重点实验室，湖北 武汉 430074; 2. State Key Laboratory of Geomicrobiology and Environmental Changes，China University of Geosciences，Wuhan 430074，China; 3.惠州港海关综合技术服务中心，广东 惠州 518060; 3. Comprehensive Technology and Service Center，Huizhou port Customs，Huizhou 518060，China; 4.重庆科技大学，复杂油气田勘探开发重庆市重点实验室，重庆 401331; 4. Chongqing Key Laboratory of Complex Oil and Gas Exploration and Development，Chongqing University of Science and Technology，Chongqing 401331，China; 5.中国科学院广州地球化学研究所，深地过程与战略矿产资源全国重点实验室，广东 广州 510640; 5. State Key Laboratory of Deep Earth Processes and Resources，Guangzhou Institute of Geochemistry，Chinese Academy of Sciences，Guangzhou 510640，China

**Keywords:** 气流吹扫注射器微萃取法, 全二维气相色谱-飞行时间质谱, 生物标志物, 原油溯源, gas purge microsyringe extraction （GPMSE）, comprehensive two-dimensional gas chromatography-time-of-flight mass spectrometry （GC×GC-TOFMS）, biomarkers, crude oil origin tracing

## Abstract

原油的化学组成极为复杂，而且各组分的相对分子质量、挥发性、含量和极性差异显著。传统的柱层析方法通常操作步骤繁琐，有机溶剂消耗大，样品处理时间长，严重限制了分析效率；常用的气相色谱-质谱（GC-MS）由于分辨率和峰容量较低，难以对复杂原油样品中的成分进行理想分离，影响了化合物的准确定性和定量分析。本研究建立了气流吹扫注射器微萃取（GPMSE）对复杂原油样品进行快速前处理的方法，在避免大量有机溶剂消耗的同时将样品处理时间缩短为10 min。结合全二维气相色谱-飞行时间质谱（GC×GC-TOFMS）技术，对45个原油样品的化学组分进行了详细分析，并构建了原油的指纹图谱。采用多元统计法对各原油样品的GC×GC-TOFMS分析数据进行处理，对不同类型化合物进行冗余分析（redundancy analysis， RDA），利用蒙特卡罗置换检验RDA排序轴的显著性，最终筛选出36个能显著反映原油来源特征的生物标志物。从45个原油样品中选取28个作为建模组构建原油来源分类模型，选取4个单一来源样品和13个混合来源样品作为验证组评估模型的有效性。结果表明该模型的判别准确率达到了97.8%。该方法不仅为原油溯源提供了高效、准确的技术支持，还具有广阔的应用潜力，可扩展至原油掺假鉴定、溢油事故责任追溯及油田开发动态监测等领域。本研究为解决原油贸易欺诈和保障国家能源安全提供了重要的技术手段，同时也为原油品质检测和风险预警提供了科学依据。

## Abstract (en)

Crude oils are complex mixtures of thousands of organic compounds that differ significantly in relative molecular mass， volatility， content， and polarity. Traditional methods for analyzing crude oil often involve complicated steps， consume large amounts of organic solvents， and require long sample-preparation times. These limitations lead to inefficient and time-consuming analysis processes. Crude oil is commonly analyzed by gas chromatography-mass spectrometry （GC-MS）. However， this technique is incapable of effectively separating complex crude-oil components owing to its low resolution and peak capacity， resulting in overlapping peaks that can lead to inaccurate compound identification and quantification. These challenges highlight the need for advanced analytical techniques. Comprehensive two-dimensional gas chromatography （GC×GC） is a novel separation technique that has been widely used to analyze complex samples， such as food， environmental samples， natural products， and crude oil. GC×GC has several advantages over traditional GC. Firstly， it offers higher resolution and peak capacity， thereby improving separation efficiency. Secondly， its high separation power reduces the need for complex sample pretreatment. Thirdly， the ordered separation and “tile effect” in a GC×GC chromatogram facilitate easier compound identification and quantification in complex mixtures.

In this study， we developed a gas purge microsyringe extraction （GPMSE） method for the rapid pretreatment of crude-oil samples. This method reduces sample processing time to only 10 min while minimizing organic solvent consumption. The chemical compositions of 45 crude oil samples were analyzed using GC×GC-time-of-flight mass spectrometry （GC×GC-TOFMS）， which helped to establish detailed chemical fingerprints for each sample. The GC×GC-TOFMS data were processed using multivariate statistical methods， including redundancy analysis （RDA） and Monte Carlo permutation testing， which identified 36 biomarkers that are strongly associated with the origin of the crude oil （p<0.05）. A classification model was constructed using a training set of 28 samples. Four single-source and 13 mixed-source samples were used to validate the model. The GPMSE-GC×GC-TOFMS method was demonstrated to be highly efficient and accurate. A discrimination accuracy of 97.8% was achieved during the identification of crude-oil sources. The developed method not only provides a powerful tool for tracing crude oil but also has broad applications potential， including for the detection of adulterated crude oil， tracking oil-spill sources， and monitoring oilfield development. This study offers several significant benefits. For example， it helps to address crude-oil trade fraud and supports national energy security. Additionally， it provides scientific support in relation to crude-oil quality control and risk assessment. The developed method is fast， reliable， and environmentally friendly； hence， it is expected to be a valuable tool for use in the oil industry. The GPMSE-GC×GC-TOFMS method is cost-effective and requires minimal solvent； consequently， it is an attractive option for reducing environmental impacts in laboratory and industrial settings. Furthermore， the high throughput and accuracy of the developed method make it suitable for large-scale analyses. In conclusion， this study demonstrated the effectiveness of combining GPMSE with GC×GC-TOFMS for analyzing crude oil； the ability of the method to identify biomarkers and classify crude-oil sources in a highly accurate manner represents a significant advancement in the field. Future studies are expected to further explore its applications in related areas， such as oil refining and environmental monitoring.

原油是我国重要的能源，年进口量突破5亿吨。为了有效监管进口原油的品质，建立高效、准确的原油溯源技术尤为重要。然而，原油及其产品的化学组成非常复杂，大约包含10 000多种有机化合物^［1，2］^，化合物的相对分子质量、挥发性、含量和极性等变化范围大^［3，4］^。目前常用的原油样品前处理方法主要是柱层析法，该方法存在操作步骤繁琐、有机溶剂用量大、样品处理时间长等不足，大大限制了原油样品的检测效率^［5，6］^。此外，一维气相色谱-质谱（1DGC-MS）因相对较低的峰容量和分辨率，很难对复杂的原油组分进行理想分离，常出现一系列共流出峰，影响了化合物的准确定性和定量^［7］^。全二维气相色谱（GC×GC）作为一种新型分离技术已广泛应用于食品^［8-11］^、环境^［12-15］^、天然产物^［16-18］^、医药卫生^［19，20］^、刑侦^［21］^、油气燃料^［22，23］^等各个领域，与1DGC相比具有如下优点^［17，22］^：第一，GC×GC具有高分辨率和峰容量，分离能力大大提高；第二，GC×GC的高分离能力可以大大简化样品前处理方法，对于复杂的石油样品只需去除非挥发性组分就可以直接进样分析，极大地提高了分析效率，而且避免了柱层析方法中低沸点组分易损失的缺点；第三，GC×GC结构谱图和“瓦片效应”有利于对化合物的准确识别和定量。

为有效应对当前进口原油所面临的严峻形势，建立简单快速的样品前处理方法及高灵敏分析方法尤为重要。本研究基于气流吹扫注射器微萃取与全二维气相色谱-飞行时间质谱（GPMSE-GC×GC-TOFMS）联用技术，对不同来源的45个原油样品中的化学组分进行了详细分析，建立了原油指纹图谱，并结合多元统计方法筛选出具有显著影响的生物标志物，可用于原油来源的鉴别。本研究建立的方法为进口原油的品质检测、产地溯源、风险预警等提供了技术支撑。

## 1 实验部分

### 1.1 仪器与试剂

GC×GC-TOFMS（Pegasus 4D）仪器由美国Leco公司生产。*n*-C_8_~*n*-C_40_烷烃混标、16种多环芳烃混标、正己烷（色谱纯）均购自上海安谱实验科技股份有限公司。原油样品由中国海洋石油集团有限公司提供，原油类型包括正常原油、轻质油和凝析油，18个原油样品来源于恩平组烃源岩，14个原油样品来源于文昌组烃源岩，13个原油样品为混合来源。

### 1.2 样品前处理及仪器分析条件

原油样品的前处理采用GPMSE法，实验装置、操作步骤及实验条件在我们前期建立的方法基础上进行了部分优化^［6］^，优化后的萃取条件如下：萃取温度为300 ℃，载气流速为2 mL/min，萃取时间为7 min，冷凝温度为‒6 ℃。

GC×GC-TOFMS柱系统由两根色谱柱组成，第一根色谱柱为非极性的DB-Petro柱（50 m×0.20 mm×0.50 μm），第二根色谱柱为中极性的DB-17HT柱（1.5 m×0.25 mm×0.15 μm），均为美国J&W Scientific公司生产。第一根色谱柱程序升温条件为50 ℃，保留3 min，以2 ℃/min升至300 ℃，保留30 min；第二根色谱柱温度比第一根色谱柱高10 ℃。调制周期为6 s；载气为氦气，纯度99.999%，流速为1 mL/min；进样口温度为300 ℃；进样量为1.0 μL；分流进样，分流比30∶1。TOFMS采集频率及采集范围为100 Hz和50~550 amu；电子轰击（EI）离子源温度为230 ℃；电离能量为70 eV；检测器电压为1.60 kV；传输线温度为280 °C。溶剂延迟6 min。

## 2 结果与讨论

### 2.1 GPMSE与传统萃取方法对比

为了验证本研究采用的样品前处理方法萃取原油样品的可靠性和优势，分别采用GPMSE法与目前原油样品前处理常用的柱层析法进行了对比。采用两种方法对同一原油样品进行前处理后再用GC×GC-TOFMS进行分析，所得结果如图1所示。从图1中可以清晰地看到，采用GPMSE法（图1a）时碳数在C_8_~C_11_的轻烃以及一些低沸点芳香烃均被有效萃取和检出，而柱层析法（图1b）中相对应的组分则完全损失或其检出信号显著降低。这种差异主要归因于以下两点：第一，柱层析法需要采用大量有机溶剂对目标组分进行洗脱，并通过氮吹或加热进行浓缩，从而导致低沸点组分的损失；第二，部分沸点或极性较高的组分未能完全从层析柱上洗脱，导致损失。总体而言，GPMSE法不仅方法操作简便、耗时短，还具有萃取效率高、对样品和有机溶剂的需求量小等优势，在原油样品前处理中具有突出优势和应用潜力。

**图1:**
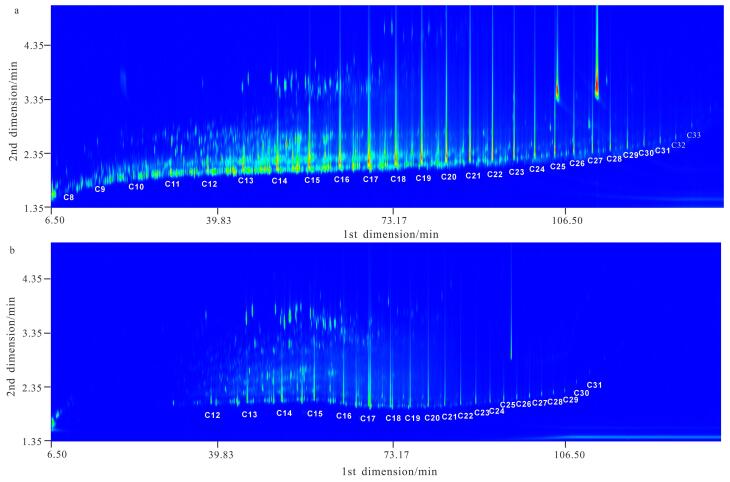
原油样品的GC×GC-TOFMS总离子流色谱图

### 2.2 原油指纹图谱的建立及原油溯源研究

首先，在1.2节的GPMSE条件下对45个不同来源的原油样品进行处理，再采用GC×GC-TOFMS对处理后的样品进行分析，得到某典型原油样品的GC×GC-TOFMS总离子流色谱图（见图1a）。采集到的GC×GC-TOFMS数据经Leco公司的软件自动处理，识别信噪比大于100的峰，再利用NIST/EPA/NIH Version 2.0标准谱图库对各色谱峰进行自动检索，检索结果生成“峰表”。对检索结果按如下程序进行人工核对：正构烷烃、多环芳烃用标准品确认定性结果，其他组分采用我们自建的原油定性数据库进行鉴定。每个样品均可鉴定出超过5 000种化合物，主要包括正构烷烃、环烷烃、藿烷、甾烷、倍半萜、双杜松烷、金刚烷、芳烃和含氮、含氧、含硫化合物等。为了进一步揭示特定化合物的特征，可选择特定质荷比（*m/z*）获得其提取离子色谱图（EIC），如图2为该原油中萜烷类（*m/z* 191）化合物的EIC谱图。基于对这45个原油样品的GC×GC-TOFMS分析结果，我们建立了原油的指纹图谱。

**图2:**
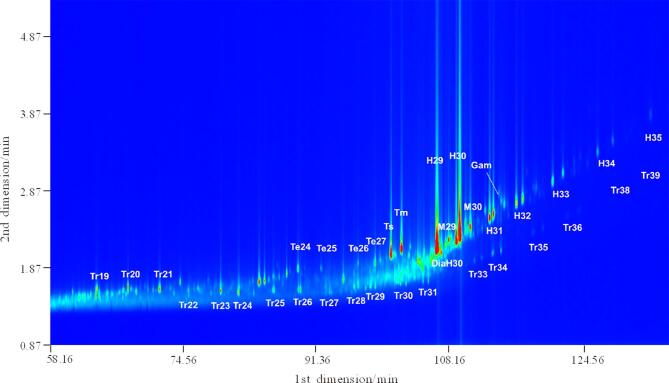
原油中萜烷类化合物的GC×GC-TOFMS提取离子色谱图（*m/z* 191）

采用多元统计法对原油样品的GC×GC-TOFMS定性定量数据进行处理，在进行统计分析前，根据公式（1）对数据进行标准化处理，将差异显著的数据归一到统一尺度上，避免因数据间的差异影响得分。


$$Z_{ij}=\frac{X_{ij}-E(X_{j})}{\sqrt[{}]{D(X_{j})}}$$ （1）

其中，*Z_ij_* 代表第*i*个样品中的第*j*个特征下的标准化值，其中*X_ij_* 是第*i*个样本在第*j*个特征下的值；*E*（*X_j_* ）是第*j*个特征的均值，*D*（*X_j_* ）是第*j*个特征的方差。

对不同类型化合物进行冗余分析（redundancy analysis， RDA），筛选原油分类参数，利用蒙特卡罗置换检验RDA排序轴的显著性，当显著性水平（*p*值）小于0.05时，可认为该参数在判断原油来源方面具有显著性。将不同的分类参数视为环境变量，根据环境变量是否通过显著性检验来确定其冗余度，筛选出能显著反映原油来源特征的生物标志物，如图3所示。

**图3:**
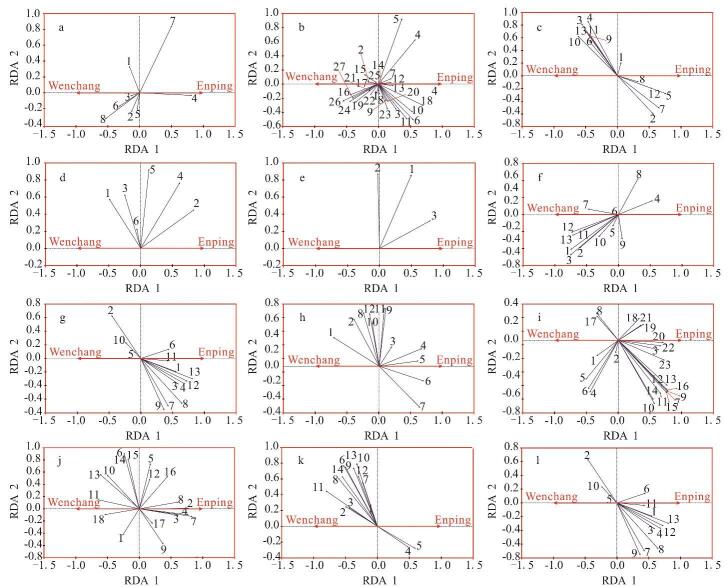
原油中不同类型标志化合物的RDA结果

基于GC×GC-TOFMS分析数据，本研究通过RDA结合蒙特卡罗置换检验（999次置换），筛选出能显著反映原油来源特征的生物标志物。参数筛选标准如下：（1）统计学显著性：蒙特卡罗置换检验的显著性水平小于0.05；（2）解释能力：RDA排序轴的解释方差占比（累计解释度）大于10%；（3）化学意义：所选参数需具有明确的生物地球化学指示意义，例如与特定烃源岩环境（如氧化/还原条件、成熟度、母质来源）密切关联；（4）独立性：通过方差膨胀因子（VIF<5）检验，避免参数间多重共线性。基于上述标准，从45个原油样品的GC×GC-TOFMS数据中筛选出36个参数，涵盖正构烷烃、萜类、甾类、芳烃及杂原子化合物等类别，构建了原油来源分类模型。通过该方法，将海量数据压缩至低维空间，且保证信息损失量尽可能少，最终建立了一个能有效识别原油来源的综合评价参数体系。

本研究采用*Q*型因子分析研究原油样品间相关关系。因子分析不仅能保证各成分之间的正交性，还能同时降低参数的维度，利于直观得出结果。利用筛选出的36个参数进行主成分分析，选取18个源自恩平组和14个源自文昌组的样品，并分为两组，每组各选取2个样品作为验证组，其余样品则为建模组（共28个样品）。首先，利用建模组样品进行模型建立，再利用验证组的4个典型样品来评估模型的有效性。最后，将13个混合来源的样品的生物标志物信息导入模型进行验证。

以建模组的28个原油样品与前面筛选出的36个参数构成28×36的矩阵 ***X*** ：


$$X=\left[\begin{array}{ccc} X_{11} & \cdots & X_{1m}\\ \vdots & \ddots & \vdots \\ X_{n1} & \cdots & X_{nm} \end{array}\right]$$ （2）

式中： $X_{ij}$ （*i*=1，2，……，*n*；*n*<28；*j*=1，2，……，*m*；*m*<36）为第*i*个样品中的第*j*个变量。为了避免因为参数间量纲的差别影响分析，对参数进行lg （*x*+1）转化处理，对转换后的相关系数矩阵进行Bartlett球度检验，所得出的相伴概率为0，小于显著性水平0.05，因此拒绝Bartlett球度检验的零假设，认为适合采用主成分分析。通过SPSS 19.0进行主成分分析，将标准化后的指标整理成矩阵 ***X*** ，并计算相关系数矩阵。通过特征值抽取方法将相关参数重新组合成一组综合参数，得到总方差解释，如表1所示，其中有8个因子的特征值大于1。第1个因子的特征值为16.28，单独解释了原始数据标准变异的45.22%；第2个因子的特征值为4.71，单独解释了13.08%；第3格因子的特征值为2.57，单独解释了7.13%。前3个因子合计解释了总信息的65.43%，基本保留了各参数所需要表达的信息。随着提取因子数量的增加，其解释份额增加不显著，因此选取前3个因子建立判别模型，得到3个判别式如下：


$$F1=0.034X_{1}-0.044X_{2}+0.047X_{3}+0.037X_{4}+0.05X_{5}+0.37X_{6}+0.44X_{7}+0.49X_{8}-0.47X_{9}+0.57X_{10}+0.024X_{11}+0.045X_{12}+0.056X_{13}+0.029X_{14}+0.003X_{15}+0.048X_{16}+0.048X_{17}+0.044X_{18}+0.047X_{19}+0.012X_{20}+0.036X_{21}+0.05X_{22}+0.043X_{23}+0.048X_{24}-0.028X_{25}-0.037X_{26}-0.053X_{27}-0.036X_{28}-0.045X_{29}+0.049X_{30}-0.054X_{31}-0.028X_{32}-0.012X_{33}-0.031X_{34}-0.015X_{35}-0.045X_{36}$$

$$F2=-0.077X_{1}+0.055X_{2}+0.048X_{3}+0.106X_{4}+0.044X_{5}+0.076X_{6}-0.061X_{7}-0.021X_{8}+0.027X_{9}-0.019X_{10}+0.0129X_{11}+0.005X_{12}-0.017X_{13}+0.148X_{14}+0.072X_{15}-0.031X_{16}+0.073X_{17}-0.018X_{18}-0.07X_{19}+0.12X_{20}+0.114X_{21}-0.057X_{22}+0.029X_{23}+0.006X_{24}+0.154X_{25}-0.025X_{26}+0.04X_{27}+0.026X_{28}-0.051X_{29}+0.095X_{30}+0.04X_{31}-0.052X_{32}+0.132X_{33}+0.135X_{34}+0.061X_{35}+0.088X_{36}$$

$$F3=-0.255X_{1}-0.074X_{2}+0.173X_{3}-0.009X_{4}-0.035X_{5}+0.208X_{6}-0.04X_{7}+0.028X_{8}+0.12X_{9}+0.007X_{10}-0.189X_{11}+0.084X_{12}-0.015X_{13}-0.095X_{14}-0.021X_{15}-0.159X_{16}+0.018X_{17}+0.32X_{18}-0.108X_{19}-0.085X_{20}-0.03X_{21}+0.081X_{22}+0.118X_{23}+0.020X_{24}-0.103X_{25}-0.076X_{26}-0.3X_{27}+0.103X_{28}-0.047X_{29}+0.075X_{30}+0.086X_{31}+0.067X_{32}-0.7X_{33}-0.081X_{34}+0.192X_{35}+0.112X_{36}$$

**表 1:** 总方差解释表

Component	Initial eigenvalues				Extract the sum of squared loads		
	Eigenvalue	Variance percent/%	Cumulative variance/%		Sum	Percent variance/%	Cumulative variance/%
1	16.28	45.22	45.22		16.28	45.22	45.22
2	4.71	13.08	58.30		4.71	13.08	58.30
3	2.57	7.13	65.43		2.57	7.13	65.43
4	2.45	6.80	72.23		2.45	6.80	72.23
5	1.67	4.63	76.87		1.67	4.63	76.87
6	1.38	3.82	80.69		1.38	3.82	80.69
7	1.18	3.28	83.96		1.18	3.28	83.96
8	1.08	3.01	86.97		1.08	3.01	86.97
……	<1						

可以发现，F1中*X*_6_~*X*_10_的系数均较大，在整个F1中占主导地位；F2中*X*_4_、*X*_14_、*X*_20_、*X*_21_、*X*_25_、*X*_33_和*X*_34_的系数均大于0.1，在整个F2中占主导地位；F3中*X*_1_、*X*_3_、*X*_6_、*X*_9_、*X*_11_、*X*_16_、*X*_18_、*X*_19_、*X*_23_、*X*_25_、*X*_27_、*X*_28_、*X*_33_、*X*_35_和*X*_36_的系数均大于0.1，在整个F3中占主导地位。

根据得分函数，对所有样品（包括验证组）进行计算，得出其F1、F2和F3值。以F1为*x*轴，F2为*y*轴，F3为*z*轴，绘制各原油样品与这3个因子的三角得分图，如图4所示。可以看出该模型将原油样品分成3类，分别代表文昌组来源、恩平组来源和混合来源。此外验证组中的2个典型文昌组来源的样品和2个典型恩平组来源的样品均落在相应区域内。在分类结果中，有1个恩平组（HZ26-1-1）来源的样品落入混合来源区域，但整体判别准确率达到了97.8%，证明了该模型的有效性。

**图4:**
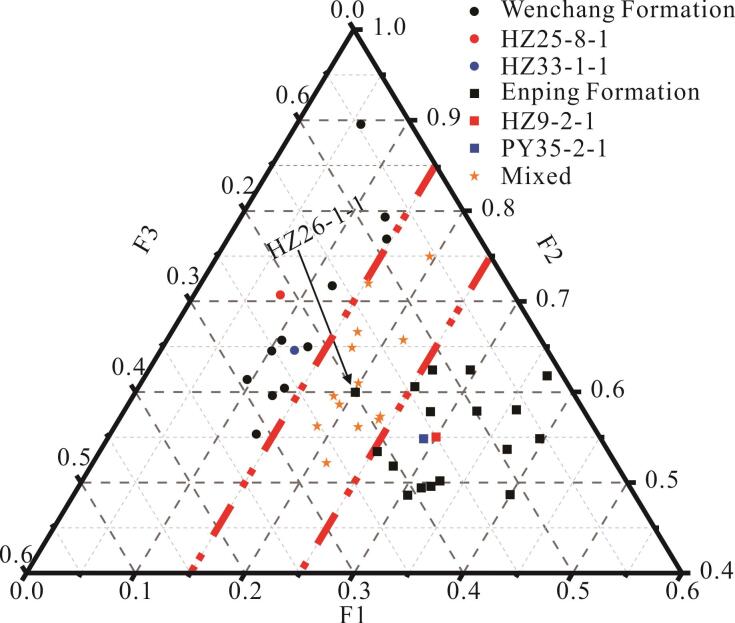
原油样品的主成分分类结果

基于上述36个生物标志物组合参数构建的综合评价体系，采用了统计学方法进行参数筛选，有效避免了传统依赖经验参数的人为主观性。该参数体系涵盖了原油中各类型化合物，能够较全面地表征原油样品的来源信息，为原油溯源研究提供了科学依据。

## 3 结论

本研究通过优化气流吹扫注射器微萃取条件，实现了复杂原油样品的高效前处理（10 min内完成，有机溶剂消耗量降至微升级），结合全二维气相色谱-飞行时间质谱联用技术，建立了高分辨原油指纹图谱分析方法。基于多元统计方法筛选的36个生物标志物参数，构建了原油来源分类模型，验证组判别准确率达97.8%，为原油溯源提供了可靠的技术支持。本文建立的方法可进一步拓展至原油掺假鉴定、溢油事故责任追溯及油田开发动态监测等领域。结合机器学习算法，有望实现多源混合原油的精准解析。当前模型基于有限样本（45个）构建，今后有必要进一步扩大样本量以增强其普适性，尤其是涵盖更多地质年代和成藏环境的原油类型；部分生物标志物（如低丰度含硫化合物）的检测受限于质谱灵敏度，未来可引入高分辨质谱（如Orbitrap）提升覆盖范围。本方法为原油溯源提供了新思路，但其实际应用需结合地质背景与多维度数据验证，后续研究将聚焦于技术标准化与跨平台数据整合，以提升方法的实用性与可靠性。
